# Telemedicine Use Among Older Adults During COVID-19: A Narrative Literature Review of Utilization Patterns

**DOI:** 10.1177/26924366251388236

**Published:** 2025-10-28

**Authors:** Meghan N. Breckling, Leah Tobey-Moore, Allie Parsons, Madeline Butera, Caraline Annichiarico, Mohab Ali

**Affiliations:** ^1^ Departments of Pharmacy Practice and Psychiatry, Center for Implementation Science, College of Pharmacy, University of Arkansas for Medical Sciences, Little Rock, Arkansas, USA.; ^2^ Departments of Psychiatry and Geriatrics, Center for Health Services Research, Psychiatry Research Institute, University of Arkansas for Medical Sciences, Little Rock, Arkansas, USA.; ^3^ College of Pharmacy, University of Arkansas for Medical Sciences, Little Rock, Arkansas, USA.; ^4^ Division of Academic Affairs, Instructor, UAMS Library, University of Arkansas for Medical Sciences, Little Rock, Arkansas, USA.; ^5^ Department of Emergency Medicine, University of Arkansas for Medical Sciences, Little Rock, Arkansas, USA.

**Keywords:** COVID-19 pandemic, digital literacy, health disparities, older adults, telemedicine, vulnerable populations

## Abstract

**Background:**

With the rapid expansion of telemedicine services during the COVID-19 pandemic, concerns have emerged about equitable access for vulnerable populations, including older adults. This narrative literature review aims to examine patterns of telemedicine use among older adults during the COVID-19 pandemic in the United States (U.S.).

**Methods:**

A comprehensive review of 55 articles published between 2020 and 2024 was conducted to analyze disparities in older adult telemedicine use around the COVID-19 pandemic. Data from electronic health records and medical claims data were compiled for analysis. Variations based on visit modalities, geographic regions and divisions, age categorization, and medical specialties were explored.

**Results:**

Most studies found lower use among older adults, with 23 reporting significantly reduced usage compared with younger groups. Only 11 showed higher use, while 12 found no difference or had inconclusive results, and 11 did not include an in-person comparison group. A total of 26 studies used a single cross-sectional design, and 29 used multiple cross-sectional designs. Research was primarily conducted in the Northeast and West, U.S., with most studies analyzing telephone, video, and in-person visits (*n* = 35) and electronic health record data (*n* = 48).

**Conclusions:**

This review reveals persistent disparities in telemedicine use among older adults during the COVID-19 pandemic, highlighting the need for research into contributing factors and targeted strategies to improve access. Policymakers should consider initiatives such as financial support, broadband expansion, and digital literacy programs to promote equity.

## Introduction

Telemedicine is the use of technology to receive medical care from a remote location. Modalities may include participating in synchronous video or telephone calls or asynchronous messaging.^1^ The COVID-19 public health emergency saw a rapid growth in the use of telemedicine services over a short period of time.^1^ Decreasing the spread of the virus by implementing stay-at-home orders was imperative, but the public still required medical care. Using CARES Act and COVID funding, many common insurances, including the Centers for Medicare and Medicaid, granted higher reimbursement rates for telemedicine services and fewer restrictions on who could utilize it.^2^ The CARES Act required Medicare to pay physicians for telehealth services at the same rate as in-office visits for all diagnoses, not just those related to COVID-19.^3^ Before COVID-19, fewer than 1% of visits used telemedicine. During the pandemic peak, this rose to 55.8–91% of encounters, then declined to an estimated 12% post-peak, still higher than pre-COVID levels.^4–6^

Telemedicine creates a unique opportunity to help reach patients who otherwise may not access medical care. It could potentially increase access to care for rural populations, decrease overall costs, and reduce travel and wait times.^2,7^ Despite the possible benefits, telemedicine is still not accessible to everyone; it may even exacerbate racial, economic, and age disparities.^2,7^ A prior review conducted by collaborating authors highlighted racial disparities in telemedicine utilization, noting that minority racial populations had lower usage rates than White patients, and African American patients were more likely to use telephone rather than video visits.^8^ Many patients may not have the appropriate resources for telemedicine services. While those with a low income could benefit from decreased transportation costs and less time off work, some barriers can still prevent them from having access to telemedicine services. They may not be able to afford a device that can perform the necessary tasks. Without the appropriate device, they are not able to consider telemedicine as an option for themselves.^9–11^ Even if obtaining a device is not a barrier, a lack of internet connectivity may raise issues for both low-income and rural patients.^9–11^ Residents living in rural areas lacking access to high-speed internet or adequate broadband may not be able to establish a connection with a health care provider to perform a virtual visit.

Another concern is the insufficient digital literacy among certain populations.^10,11^ One proposed definition of digital literacy is the ability to access, manage, understand, integrate, communicate, evaluate, and create information safely and appropriately through digital technologies.^12^ Populations that may not have the experience or knowledge include racial minorities, the unemployed, and older adults; many of these are the same populations that could benefit from telemedicine. Older adults may struggle with digital tools because of limited exposure to newer technologies. Having grown up before the current wave of rapid technological change, they may find it difficult to stay up to date. Older adults may also be low-income, which, as previously discussed, can limit their access to technology.

People aged 65 and older are more likely to struggle with using digital technology and are considered a vulnerable group. With the increasing use of telemedicine throughout the medical field, older adults are at risk of being left behind in terms of access to care.^2^ Although telemedicine expanded rapidly during COVID-19, research offers limited insight into specific factors of older adults' use and geographic disparities, motivating this review. This narrative review aims to describe trends in telemedicine utilization among older adults in the United States (U.S.), accounting for variations in visit modality, geographic region and division, age classification, and type of specialty care.

## Methods

### Literature search

A medical librarian (Annichiarico) performed an electronic search of the literature on January 11, 2024, using the following databases: MEDLINE via OVID, Embase, and Web of Science. Expert searching techniques included the use of medical subject headings, text words, truncation, wildcards, nesting, and adjacency searching. The search strategy incorporated search terms based on the concepts of telemedicine, COVID-19, outpatients, and disparities. The search terms were adjusted accordingly to fit each appropriate database. See Supplementary Data for MEDLINE search strategy. Language was limited to English, and no date restrictions were applied. The search strategy was peer-reviewed by a second medical librarian.

### Eligibility criteria

Inclusion criteria consisted of synchronous visits, data from electronic medical records, medical claims data, completed visits, and studies conducted within the U.S. health care system. Exclusion criteria consisted of telemedicine services provided to pediatrics, people 17 years or younger, and veterans. Study characteristics excluded were asynchronous visits, virtual hospitals, e-messaging through patient portals, data collected via surveys, incomplete visits, and studies conducted outside of the U.S. Asynchronous visits were excluded because reimbursement and health care policies during and after COVID-19 focused mainly on synchronous care, making disparities in its use more relevant to policy and practice. Study design types excluded were systematic reviews, scoping reviews, conference proceedings, clinical trials, retrospective chart reviews, and qualitative or prospective studies.

### Study selection

Citations were uploaded to Covidence, an online screening tool, where duplicates were identified and removed. Three authors (B.M.N., T.-M.L., and A.M.) independently screened titles and abstracts using the specified inclusion and exclusion criteria stated above. A third reviewer was utilized to resolve conflicts if consensus could not be reached. Full texts of eligible articles were independently assessed (P.A. and B.M.) and excluded based on reasons of ineligible publication type, study design, outcomes, setting, and absence of age data. See the Supplementary Data for the Preferred Reporting Items for Systematic reviews and Meta-Analyses (PRISMA) flow diagram. Authors (B.M.N., T.-M.L., and A.M.) devised data points in an Excel spreadsheet, and when articles met the inclusion criteria, data were extracted and recorded.

#### Identifying patterns

After extraction, the included studies were split up into two tables for comparison of articles with a single cross-sectional cohort with articles comparing two or more cross-sectional cohorts, pre- and post-COVID. Based on the location of each article’s included population, U.S. region and U.S. division categories were assigned based on published U.S. Census Codes.^13^ The Census divides the U.S. into four regions, which are further subdivided into nine divisions.^13^ Final summary tables for this review included the type of data (electronic health record [EHR] or claims data), units of analysis (patients or visits), sample size, telemedicine modality (video and/or phone), if in-person visits were included, how age was collected, and whether it was reported that older adults used telemedicine more, less, no difference in use, or if there was inconclusive results. Older adult telehealth utilization patterns included those 50 years and above. Articles were ordered in Tables 1 and 2 based on the reported results of telemedicine use in older adult age groups. Since articles reported age in a variety of ways (mean, median, average, age groups), the reported age variables were included in the tables.

**Table 1.** Single Cross-Sectional Studies on Telehealth Use in Older Adults

*YOP*	*Author*	*U.S. region*	*U.S. division*	*Data type*	*Unit of analysis*	*Sample size*	*Modalities*	*Age variable*	*Older adult telehealth utilization*
2022	Frydman et al.^14^	1	2	EHR	Patients visits	491 1,783	Telephone, video, in-person	Age groups: >65, ≤65	Less telemedicine use
2022	Nguyen et al.^15^	3	5	EHR	Patients	198	Telephone, video, in-person	Mean age	Less telemedicine use^a^
2022	Kubes et al.^16^	3	5	EHR	Patients	18,148	Video, In-Person	Age groups: <18, 18–34, 35–64, 65+	Less telemedicine use^a^
2022	Haggerty et al.^17^	3	5	EHR	Visits	110,999	Telephone, video, in-person	Age groups: 35–64, 65+	Less telemedicine use^a^ *Except older, sicker patients may increase use.*
2023	Osmanlliu et al.^4^	4	9	EHR	Visits	58,940	Telephone, video, in-person	Age groups: 60–79, >/= 80	Less telemedicine use^a^ audio only > video^a^
2023	Cherabuddi et al.^18^	2	3	EHR	Visits	72,153	Telephone, video, in-person	Age groups: 18–29, 30–49, 50–65, and >65 years	Less telemedicine use^a^ audio only > video^a^
2021	Haynes et al.^19^	4	9	EHR	Patients	1,292	Telephone, video, in-person	Age groups: 1–24, 25–49, 50–65, >65	Less telemedicine use.^a^
2023	Ostovari et al.^20^	1, 3	2, 5	EHR	Patients	41,583	Telephone, Video, In-Person	Average Age	Less telemedicine use.^a^ audio only > video
2021	Wang et al.^21^	1	1	EHR	Patients visits	10,113 11,394	Telephone, video, in-person	Age groups: <66, 61–75, >75	Less video telemedicine use^a^
2022	Elam et al.^22^	2	3	EHR	Patients	1,720	Telephone, video, in-person	Mean age	Less video telemedicine use^a^
2022	Chen et al.^23^	1	1	EHR	Patients	5,023	Telephone, video, in-person	Median age	Less video telemedicine use^a^ audio > video^a^
2023	Tobin et al.^24^	2	3	EHR	Visits	1,075	Telephone, video, in-person	Mean age	More audio only telemedicine use^a^ audio only > video^a^
2021	Hsiao et al.^25^	2	3	EHR	Patients visits	197,076 504,464	Telephone, Video, In-Person	Mean age	More audio telemedicine use.^a^
2023	Hsiao et al.^26^	2	3	EHR	Patients visits	420,876 2,494,296	Telephone, video, in-person	Mean age	More telemedicine use^a^ audio only > video^a^
2021	Rodriguez et al.^27^	1	1	EHR	Patients visits	162,102 231,596	Telephone, video, In-Person	Age groups: 18–44, 45–64, 65+	More telemedicine use. audio only > video^a^
2023	AbouAli et al.^28^	1	2	EHR	Patients visits	10,587 23,553	Telephone, Video, In-Person	Mean Age	No difference
2022	Waseem et al.^29^	3	5	EHR	Patients visits	720 2,031	Video, in-person	Age groups: >65, ≤65	No difference
2022	Zhu et al.^30^	4	9	EHR	Visits	9,332	Video, in-person	Age groups: 16–29, 30–39, 40–49, 50–59, 60–69, 70–79	No difference
2022	Govier et al.^31^	4	8, 9	EHR	Patients	11,326	Telephone, video, in-person	Age groups <20, 20–34, 35–44, 45–54, 55–64, 65–74, and ≥75	No difference
2022	Hossain et al.^32^	3	5	Medicaid claims	Patients	320,648	Telephone, video	Age groups 0–18, 19–64, 65+	*No in-person group* audio only > video
2022	Odukoya et al.^33^	2	3	Claims-based	Patients	4,744	Telephone, video	Age groups 0–18, 19–64, 65+	*No in-person group* audio only > video^a^
2021	MyersVirtue et al.^34^	1	1	EHR	Patients	354	Telephone, video	Mean age	*No in-person group* audio only > video^a^
2021	Strowd et al.^35^	3	5	EHR	Visits	1,011	Telephone, video	Mean age	*No in-person group* audio only > video^a^ *(no in-person group)*
2020	Eberly et al.^36^	1	2	EHR	Patients	148,402	Telephone, Video	Age groups <55, 55–64, 65–74, 75+	*No in-person group* less completed telemedicine visits^a^ audio only > video^a^
2021	Poeran et al.^37^	n/a	n/a	Commercial Claims	Patients visits	46,421,994 846,461,609	Telephone, Video	Age groups <54, 55+	*No in-person group* less telemedicine use^a^
2020	Eberly et al.^38^	1	2	EHR	Patients	2,940	Telephone, Video	Mean age	*No in-person group* more completed telemedicine visits^a^

The Census divides the U.S. into four regions, which are further subdivided into nine divisions.^13^

Studies grouped based on “Older Adult Telehealth Utilization.”

^a^ Statistically significant outcome.

EHR, electronic health record; YOP, Year of Publication; U.S., United States.

**Table 2.** Multiple Cross-Sectional Studies on Telehealth Use in Older Adults

*YOP*	*Author*	*U.S. region*	*U.S. division*	*Data type*	*Unit of analysis*	*Sample size*	*Modalities*	*Age variable*	*Older adult telehealth utilization*
2021	Chunara et al.^39^	1	2	EHR	Visits	494,317	Telephone, video, in-person	Mean age	Inconclusive
2021	George et al.^40^	Multiple	Multiple	EHR	Patients visits	126,550 303,037	Telephone, video, in-person	Age groups: <65, 65+	Inconclusive fewer in-person and telehealth visits for those ≥65 years.
2023	Buis et al.^41^	2	Multiple	EHR	Patients visits	178,747 938,040	Telephone, video, in-person	Age groups: 18–64, 65+	Inconclusive Fewer in-person visits for older adults (≥65 years). No difference in video or phone visits for older adults (≥65 years).
2022	Neeman et al.^42^	4	9	EHR	Visits	334,666	Telephone, video, in-person	Age groups: Gen Z 1997+, Millennials/Gen Y 1982–1996, Gen X 1965–1980, Baby Boomers 1946–1964, Silent Generation before 1946	Inconclusive Less video and in-person use for older adults. More telephone use for older adults.
2023	Shah et al.^43^	4	8	EHR	Patients visits	164,647 311,517	Telephone, video, in-person	Age groups: 18–24, 25–34, 35–44, 45–54, 55–64, 65+	Less telehealth use for age 55 and over.
2023	Chen et al.^44^	1	2	EHR	Patients	190,949	Telephone, video, in-person	Age groups: 18–44, 45–64, 65+	Less telehealth use for age over 65.^a^
2023	Frontera et al.^45^	2	3	EHR	Patients	4,909	Telephone, video, in-person	Single age group: 65 or older	Less telehealth use for age over 65.^a^
2023	Rockholt et al.^46^	1	2	EHR	Patients	12,615	In-Person, telemedicine (undefined)	Age groups: 0–19, 20–29, 30–39, 40–49, 50–59, 60–69, 70–79, 80–89, 90–99, >100, missing	Less telehealth use for age over 70.^a^
2021	Sachs et al.^47^	4	9	EHR	Patients	134,274	Telephone, video, in-person	Age groups: 0–17, 18–34, 35–64, 65+	Less telehealth use in age over 65
2023	Palzes et al.^48^	4	9	EHR and claims-based	Patients	36,607	Telephone, video, in-person	Age groups: 18–34, 35–49, 50–64, 65+	Less telehealth use in age over 65^a^
2022	Eruchalu et al.^49^	1	1	EHR	Visits	4,908	Telephone, video, in-person	Median age	Less telemedicine use in older adults^a^ audio > video for older adults^a^
2022	Kummer et al.^50^	1	2	EHR	Patients	14,170	Video and in-person	Median age	Less telemedicine use^a^
2021	Yuan et al.^51^	4	9	EHR	Visits	176,781	Telephone, video, in-person	Mean age	Less video use in older adults^a^
2022	Sanaka et al.^52^	2	3	EHR	Visits	46,041	Telephone, video, in-person	Mean age	Less video use in older adults^a^
2023	Park et al.^53^	3	7	Claims-based	Patients	850,821	In-person, telemedicine (undefined)	Age groups: 0–17, 18 –34, 35–49, 50–64	More telehealth use for 50–64 years. No statistical values provided for significance.
2021	Qian et al.^54^	4	9	EHR and claims-based	Patients	4,556,646	Telephone, video, in-person	Age groups: 0–5, 6–17, 18–44, 45–64, ≥65	More telehealth use in adults age 65 and older.
2023	Zachrison et al.^55^	1	1	EHR	Patients	1,241,313	Telephone, video, in-Person	Age groups: <50, 50–64, 65–85, 85+	More telehealth use in age over 50^a^; audio > video^a^
2022	Annapragada et al.^56^	3	5	EHR	Patients	12,163	Telephone, video, in-person	Age groups: <27, 27–54, 54–82, 82+	More telehealth use in age over 54^a^
2021	Stevens et al.^57^	1	1	EHR	Visits	310,675	Telephone, video, in-person	Age groups: 18–29,30–39,40–49,50–59,60–69,70–79, >80	More telehealth use in older patients >70^a^ audio > video
2021	Esper et al.^58^	3	5	EHR	Patients	7,119	Telephone, video, in-person	Mean Age	More telemedicine use for age 60–79 years. audio > video for older adults^a^
2023	Sanchez et al.^2^	3	7	EHR	Patients visits	293,877 374,803	Telephone, video, in-person	Age groups: 18–44, 45–54, 55–64, ≥65	More use of telephone visit for age 65 and older^a^
2021	Tsao et al.^59^	4	9	EHR	Patients visits	382 Total 967 total	Telephone, video, in-person	Median age	No difference
2024	Ye et al.^60^	3	5	EHR	Patients	8,197	In-person, telemedicine (undefined)	Single age group: >65	No difference.
2020	Lonergan et al.^61^	4	9	EHR	Patients	15,230	Video	Median age	No difference.
2021	Lattimore et al.^62^	3	5	EHR	Patients visits	14,792 21,980	Telephone, video	Mean age	No difference.
2023	Shao et al.^63^	3	7	Claims-based data	Patients	51,817	Telephone, video	Age groups: 18–39, 40–54, 55–64	*No in-person group* less telehealth use for age 55–64.^a^
2022	Datta et al.^64^	2	4	EHR	Patient	252,453	Telephone, video	Age groups: < 19, 19–34, 35–49, 50–64, 65–79, 80+	*No in-person group* less telehealth use for age over 65.^a^
2022	Rini et al.^65^	4	9	EHR	Visits	34,000	Telephone, video	Mean age	*No in-person group* more telehealth use in older adults.^a^
2022	Pagan et al.^66^	4	9	EHR	Patients	19,376	Telephone	Age groups: <18, 18–64, 65+	*No in-person group* more telephone use in older adults (65 and older).^a^

The Census divides the U.S. into four regions, which are further subdivided into nine divisions.^13^

Studies grouped based on “Older Adult Telehealth Utilization.”

^a^ Statistically significant outcome.

EHR, electronic health record; YOP, Year of Publication; U.S., United States.

## Results

### Findings from single cross-sectional studies on Telemedicine use in older adults

Among the 55 studies that met the inclusion criteria, 26 used a single cross-sectional design, analyzing data collected from a single time point (Table 1). Sample sizes varied widely across these studies, from as few as 198 patients across 1,011 visits to as many as 420,876 patients over 846 million visits. Most studies (23 of 26) utilized data from electronic health records (EHRs), while 3 used insurance claims data.

Geographically, the largest number of studies were conducted in the Northeastern U.S. (9 studies), with the fewest in the Western region (4 studies). Regarding age classification, 15 studies use age group clusters, 9 reported mean age, and 2 reported either average or median age. The predominant trend across these studies was lower telemedicine utilization among older adults. Specifically, 11 studies reported significantly reduced use of telemedicine in older populations compared with younger groups. In contrast, only 4 studies reported higher telemedicine usage among older adults, while 11 studies reported either no difference or inconclusive results or lacked the inclusion of in-person visits as a comparison.

### Findings from multiple cross-sectional studies on Telemedicine use in older adults

The remaining 29 studies used a multiple cross-sectional design, drawing on data collected at more than one point in time (Table 2). These studies also demonstrated a wide range in sample sizes, from 1,766 patients across 1,915 visits to 1,241,313 patients across 4,556,646 visits. Most studies (25 of 29) relied on EHR data, while 2 studies used insurance claims data, and 2 studies combined both sources.

Regionally, the Western U.S. was the most common study location (11 studies), whereas the Midwest hosted the fewest (4 studies). In terms of age categorization, 19 studies used age cluster groupings, 6 studies reported mean age, and 4 reported median age. As with the single cross-sectional studies, most of the findings indicated reduced telemedicine use among older adults. A total of 12 studies reported significantly less use in older populations, 7 reported greater telemedicine usage, and 12 showed either no significant difference, inconclusive results, or a lack of direct comparisons with in-person care.

### Geographic distribution of included studies

The included studies were distributed across the four U.S. Census regions, with noticeable variation in representation. Of the single cross-sectional studies (*n* = 26), the majority were conducted in the Northeast (9 studies), followed by the South (7), Midwest (6), and West (4). Within U.S. divisions, the Middle Atlantic (5), South Atlantic (6), and East North Central (6) divisions were the most represented. Notably, several divisions, West North Central and West South Central, had no representation at all. In contrast, of the multiple cross-sectional studies (*n* = 29), most were conducted in the West (11 studies), with fewer in the South (7), Northeast (7), and Midwest (4). The Pacific division alone accounted for 10 of the 11 studies in the Western region, highlighting a concentration of research activity within this specific study design.

### Visit modality types assessed

The studies varied in the combinations of visit modalities they assessed, ranging from in-person comparisons with audio-visual telemedicine only. Within the single cross-sectional studies, most studies included telephone, video, and in-person visits (16 studies), while others examined combinations such as telephone and video (7 studies) or video and in-person (3 studies). Notably, no studies assessed video-only modalities. Alternatively, there was more variability among the multiple-cross sectional studies; most included telephone, video, and in-person visits (19 studies), while others were more limited: telephone and video (4 studies), video and in-person (1 study), telephone only (1 study), and video only (1 study). A few studies compared in-person with telemedicine without a specific modality breakdown (3 studies). Across both single and multiple cross-sectional studies, those that showed more telemedicine use in older adults primarily saw higher telephone (audio) use compared with video.

### Types of clinical settings represented

Although not explicitly categorized in all included studies, the clinical settings spanned a wide range of health care domains. Based on the studies reviewed in Tables 1 and 2, most were situated in primary care, internal medicine, or subspecialty care settings, with a strong emphasis on telemedicine for chronic disease management (e.g., cardiology, endocrinology, and geriatrics). Several studies also involved ambulatory care clinics, particularly those affiliated with academic medical centers or large health systems. However, given the inconsistent reporting of clinical settings, a uniform quantitative analysis was not feasible, and the findings are instead summarized descriptively.

## Discussion

This narrative literature review aimed to explore age-related patterns in telemedicine utilization during the COVID-19 pandemic, with a specific focus on older adult populations. Although we did not conduct a formal systematic review or meta-analysis, our structured and methodical approach, facilitated by the Covidence platform, enabled us to identify recurring themes and disparities in telemedicine access and usage by age group across 56 U.S.-based studies.

### Interpretation of findings

Across both single and multiple cross-sectional designs, a consistent pattern emerged: older adults were less likely to use telemedicine services compared with younger individuals. Specifically, more than 40% of the studies in each design category reported lower telemedicine usage among older populations. These findings align with prior literature citing digital literacy, technology access, and comfort with virtual care platforms as major barriers to telemedicine adoption in older adults.^10,11^ In contrast, a smaller proportion of studies observed higher use or no significant difference, suggesting that the gap may be closing in certain clinical contexts or health care systems that have invested in older adult telemedicine engagement strategies or campaigns.

Importantly, this analysis draws on real-world data, including EHRs and insurance claims, offering a view into actual telemedicine utilization rather than self-reported intentions or preferences. However, heterogeneity in study settings, populations, and outcome measures makes it difficult to generalize findings universally. For example, geographic analysis revealed a concentration of studies in the Northeast and Western U.S. regions, with limited studies in the Midwest region, potentially limiting national representation.

### Clinical setting and modality considerations

While studies spanned a variety of clinical settings, such as primary care, subspecialty clinics, and academic health systems, reporting across all included studies was inconsistent. This variation limits the analysis of the specific use of different modalities and implementation strategies. Most studies examined a mix of telephone, video, and in-person visits, though relatively few focused on video-only interactions, which might require better access to technology and higher digital proficiency, factors that can be especially challenging for the older adult population. This review indicates that in studies where older adults used more telemedicine, the telephone (audio-only) was the most used modality compared with video. Similarly, a comprehensive review found that studies including audio visits reported reduced racial disparities in telemedicine use.^8^ Telephone visits may provide a more accessible entry point for this population but may also limit the clinical thoroughness of assessments and reimbursement options with various insurance plans. Under the current Centers for Medicare and Medicaid Services (CMS) policy, video-based telemedicine is expected to remain widely reimbursable across most payers. In contrast, coverage for audio-only visits remains more limited and inconsistent, with long-term reimbursement still under evaluation in many regions. If CMS adopts a policy to permanently cover audio-only visits, other payers, such as private insurers, may follow, helping to reduce barriers and expand telemedicine access for vulnerable populations, such as older adults.

### Implications for access and equity

The rapid expansion of telemedicine during the pandemic was intended to preserve care continuity, but it may have unintentionally amplified existing age-related disparities. Older adults often face intersecting barriers, such as lower digital literacy, reduced digital access, especially in rural regions, and limited income, that collectively hinder equitable participation in telemedicine. Our review reinforces the need for tailored strategies that account for the challenges many older populations face. Interventions (e.g., educational campaigns) could include simplified telemedicine platforms, technical support hotlines, caregiver engagement, or hybrid care models that blend in-person and virtual options based on patient capability.

### Considerations and limitations

As outlined in our methods, we intentionally focused on U.S.-based, quantitative, synchronous visit studies using structured data sources. While this provided consistency, we recognize it also introduced limitations. For instance, by excluding qualitative and survey-based studies, we may have missed nuanced insights into patient attitudes and barriers among older adult populations. The heterogeneity in how age and visit modality were reported in the included studies also limited opportunities for meta-level statistical analysis. Furthermore, due to inconsistent reporting of clinical settings by the studies, we were unable to draw quantitative comparisons across case settings and thus presented these descriptively.

### Future directions

Building on the findings of this review, future research should move beyond documenting disparities to identifying and testing interventions that improve telemedicine engagement and access among older adults. Mixed-methods studies that integrate quantitative telemedicine usage patterns with nuanced qualitative insights from patients and providers could offer a more comprehensive understanding of the barriers many older adults face. In addition, evaluating various implementation strategies such as simplified digital interfaces, telemedicine educational campaigns, caregiver-supported models, and tailored onboarding programs may help determine what works best to engage older adults in different U.S. regions and varying care settings. Longitudinal studies are also needed to assess the long-term effects of expanded telemedicine access on health outcomes in aging populations. Finally, factors such as income, race, location, and health influence telemedicine access and should guide future efforts to support the diverse needs of older adults as virtual care becomes permanent.

## Abbreviations Used

AI: Artificial Intelligence
CARES Act: Coronavirus Aid, Relief, and Economic Security Act
ChatGPT: Chat Generative Pre-trained Transformer
CMS: Centers for Medicare and Medicaid Services
COVID-19: Coronavirus Disease 2019
EHR: Electronic health record
PRISMA: Preferred Reporting Items for Systematic Reviews and Meta-Analyses
U.S.: United States
