# Supplementary material for: Telemedicine Use Among Older Adults During COVID-19: A Narrative Literature Review of Utilization Patterns
Source: Telemed Rep. 2025 Oct 28;6(1):371–81. doi: 10.1177/26924366251388236 (PMC12670650; doi:10.1177/26924366251388236)
Supplement: Supplementary Data [file 26924366251388236_suppl_data.docx]

Studies from databases/registers **(n = 570)**

Web of Science (n = 206)

Ovid MEDLINE (n = 191)

Embase (n = 173)

**Identification**

Studies included in review **(n = 55)**

Studies excluded **(n = 263)**

Studies assessed for eligibility **(n = 169)**

Studies sought for retrieval **(n = 169)**

Studies screened **(n = 432)**

Studies excluded **(n = 114)**

Wrong Setting (n = 1)

Wrong Outcomes (n = 2)

Wrong Population (n = 14)

Wrong Study Design (n = 18)

Wrong Publication Type (n = 48)

Age disparity not included in study (n = 30)

Miscellaneous Exclusion (n = 1)

References removed **(n = 138)**

**Screening**

**Included**
